# Functional Molecular Network Analysis Enables Prediction of Response to Vedolizumab Therapy in Anti-TNF Refractory IBD Patients

**DOI:** 10.1093/crocol/otaa037

**Published:** 2020-05-24

**Authors:** Matthias Breidert, Pierre Eftekhari, François Louis, Claudia Rotoiu, Timo Rath, Markus F Neurath, Raja Atreya

**Affiliations:** 1 Inoviem Scientific, BIOPARC 3, Illkirch-Graffenstaden, France; 2 Department of Gastroenterology and Hepatology, City Hospital Waid and Triemli, Zürich, Switzerland; 3 Department of Medicine I, Friedrich-Alexander-Universität Erlangen-Nürnberg, Erlangen, Germany; 4 Deutsches Zentrum Immuntherapie (DZI), Friedrich-Alexander-Universität, Erlangen-Nürnberg, Erlangen, Germany; 5 The Transregio 241 IBDome Consortium

**Keywords:** vedolizumab, inflammatory bowel disease, physiological intermolecular modification spectroscopy (PIMS), nematic protein organization technic (NPOT), responder interactors

## Abstract

**Background:**

We applied for the first time 2 label-free technologies, physiological intermolecular modulation spectroscopy (PIMS) and nematic protein organization technic (NPOT) in anti-tumor necrosis factor (TNF) refractory inflammatory bowel disease (IBD) patients to identify clinical responders to vedolizumab therapy and elucidate their underlying functional molecular network.

**Methods:**

PIMS analysis was performed in peripheral blood taken prior to the first vedolizumab application in 20 IBD patients (Crohn disease n = 13; ulcerative colitis n = 7) refractory to at least 1 previous anti-TNF agent therapy. Peripheral blood taken from clinical responders and nonresponders at week 14 of vedolizumab therapy were additionally subjected to NPOT analysis. Response to therapy was assessed by respective clinical disease activity scores (partial Mayo Score and Harvey–Bradshaw Index).

**Results:**

Clinical response to vedolizumab treatment was observed in 7 of 13 Crohn disease and 4 of 7 ulcerative colitis patients at week 14. Response to therapy was accurately predicted by PIMS blood analysis in 100% of ulcerative colitis and 77% of Crohn disease patients. Overall prediction of clinical response with PIMS blood analysis was achieved with a 89% positive predictive value and a 82% negative predictive value. NPOT analysis revealed the heightened expression of the proteins ITGB7, ITGAV, ITG3, PF4, and ASGH in the peripheral blood of vedolizumab responders compared to nonresponders.

**Conclusions:**

PIMS analysis of the blood of anti-TNF refractory IBD patients was able to stratify responders to vedolizumab therapy with high accuracy and specificity. NPOT technology could decipher underling molecular networks in the blood of responders, enabling subsequent personalized therapeutic approaches in IBD.

## Lay Summary

Two novel technological methods (physiological intermolecular modulation spectroscopy and nematic protein organization technic) were used to analyze the blood of inflammatory bowel disease patients, and allowed us to predict the clinical effectiveness of the therapeutic antibody vedolizumab in the treatment of these patients.

## INTRODUCTION

Ulcerative colitis and Crohn disease are the major entities of inflammatory bowel diseases (IBD). Both are chronic inflammatory diseases, with possible acute exacerbations, upon which patients suffer from inflammation of the large or small intestine. The annual incidence of IBD is 3–8.5/100,000 in European countries and as many as 2.2 million individuals in Europe and 1.4 million individuals in the United State suffer from IBD.^1^ Both forms of IBD are associated with marked morbidity and have a major impact on an individual’s quality of life and their ability to work, which highlights the need for optimized anti-inflammatory therapy.^2^

Recent advances in understanding the underlying immunopathogenetic mechanisms of IBD have led to the development of biological therapies, which selectively inhibit crucial mediators of the inflammatory process. First, tumor necrosis factor (TNF) was identified as one of the central proinflammatory cytokines, which resulted in the development of antibodies that neutralize the biological activity of TNF and demonstrated therapeutic efficacy in inducing and maintaining remission.^3^

Due to the inflammatory process it seems that immune cell migration is increased and thus this mechanism was addressed with the development of anti-integrins.^4^ Vedolizumab, a humanized monoclonal antibody directed against the alpha4beta7 integrin has been approved for treatment of ulcerative colitis and Crohn disease, as it demonstrated therapeutic efficacy in inducing and maintaining remission. However, across all trials about 40% of patients do not respond to treatment with vedolizumab. Furthermore, in comparison to anti-TNF antibody naive patients, there is a diminished response rate in patients that have previously been exposed to inefficient anti-TNF therapy.^5,6^ A central aspect of the management of these patients is therefore to establish clinically applicable predictive markers to ensure a rapid therapeutic benefit in treated vedolizumab patients, reduce potential side effects of an inefficient therapy and ensure health-economic sound utilization of this therapeutic approach. These predictive markers should be utilized to identify patients with high probability of response before initiation, to help make a decision to continue or discontinue therapy.^7^

So far, only a limited number of potential predictive markers have been identified in recent research proceedings but mechanisms of nonresponse to vedolizumab are still incompletely understood. From a disease-related perspective, it appears that patients with less severe disease without prior anti-TNF exposure and who demonstrate early response to vedolizumab therapy have the highest probability of achieving durable clinical response and remission.^8–10^ Another study indicated that baseline community alpha-diversity was significantly higher, and *Roseburia inulinivorans* and *Burkholderiales* species more were abundant among patients with Crohn disease who achieved remission at week 14 of vedolizumab treatment compared to nonremitters.^11^ A recent small pilot study, using molecular imaging of alpha4beta7 integrins suggested that pretherapeutic low mucosal integrin expression was associated with primary nonresponse to vedolizumab in Crohn disease.^12^ However, validation studies are needed and translation of these findings into clinical practice has not yet been achieved.^7^

To the best of our knowledge, there have so far not been any proteomic based approaches for the identification of predicting responders to subsequent vedolizumab therapy. Recent technical advances, eg, in the field of proteomics, subproteomic, or metabolomics have gained marked interest, giving new hope for biomarker identification in the field of IBD. Proteomic and subproteomic analyses have additionally identified a large number of different proteins that are overexpressed in IBD patients dependent on the methodological approach, but currently these insights have not been studied regarding prediction of therapeutic vedolizumab response.^11^

Physiological intermolecular modulation spectroscopy (PIMS) is a patented label-free technology (WO2013139988 (A1)). It takes into account a combination readout based on changes in the resonance of water molecules and macromolecular conformation. The latter can be used to predict treatment efficacy. Altogether, PIMS provides significant opportunities in the field of personalized medicine and biomarker development. We have already applied PIMS technology to accurately predict response to subsequent anti-TNF therapy in 30 IBD patients.^13^

Nematic protein organization technic (NPOT) is a label-free technology able to identify clinical mode of action of a compound or pharmacologically active agent directly from human tissue. NPOT is particularly effective for identifying therapeutic (ON) or toxic (OFF) targets of a molecule by enabling the label-free formation of macromolecular protein scaffolds, containing the exhaustive list of complexes involved in physiological or pathological processes.^14^

The objective of our study was to determine the spectral characteristic of vedolizumab-treated IBD patient’s macromolecular assemblies of peripheral blood cells and of the specific spectra with PIMS and NPOT to enable stratification of anti-TNF refractory IBD patients into responders or nonresponders to subsequent vedolizumab therapy. This approach could enable a more personalized therapeutic approach in IBD patients in the future.

## MATERIALS AND METHODS

### Patient Characteristics

This was a longitudinal study that was performed at the IBD outpatient Clinic of the Medical Department 1 of the University of Erlangen-Nürnberg, Germany. Twenty consecutive anti-TNF refractory IBD patients (13 Crohn disease and 7 ulcerative colitis) that matched the inclusion and exclusion criteria and agreed to participate were included in this study. All patients previously demonstrated nonresponse to at least one of the approved agents (infliximab, adalimumab, certolizumab pegol, or golimumab) for the treatment of ulcerative colitis or Crohn disease. Majority of patients were nonresponsive to at least 2 different anti-TNF substances (17 of 20 analyzed patients). Nonresponse was determined by discontinuation of previous anti-TNF antibody treatment due to therapeutic inefficiency (primary or secondary nonresponse). There were no anti-TNF trough or antidrug antibody measurements available for the studied patients.

The inclusion criteria for patients aged between 18 and 75 years was proven diagnosis of one of the disease entities (>3 months) based on clinical, endoscopic, and histological criteria. Patients had to have moderate to severe clinical active ulcerative colitis [partial Mayo Score ≥5] or Crohn's disease [Harvey–Bradshaw Index (HBI) score ≥8] at baseline and indication for the initiation of vedolizumab therapy. For patients with ulcerative colitis, the partial Mayo Score, which ranges from 0 to 9, was used with the known noninvasive components (stool frequency, rectal bleeding, and Physician’s global assessment). This simplified index maintains a rather good relationship with the full Mayo Score in identifying clinical response as perceived by patients.^15^ For clinical response, a reduction of the partial Mayo Score of at least 3 points and at least 30% vs baseline, which includes a decrease in the score for rectal bleeding of at least 1 point, or an absolute score for rectal bleeding not exceeding 1 at week 14 of ongoing vedolizumab therapy was used.^16^ For Crohn disease patients, clinical response was defined as a reduction of 3 or more points of the HBI score^17^ at week 14 in comparison to baseline before the start of vedolizumab therapy.^18^ A 3-point reduction in the HBI score was described to correspond to a 100-point change in the Crohn’s Disease Activity Index, and was therefore chosen as the threshold for clinical response in our study.^19^ There was no inclusion of endoscopic findings in both disease entities, as there was no obligatory endoscopy performed in this study prior or during the course of vedolizumab therapy. Concomitant medication included 5-aminosalicylates, budesonide, corticosteroids, methotrexate, 6-mercaptopurine, or azathioprine. Immunosuppressive (thiopurines and methotrexate) therapy had to be present in unchanged dosing at a minimum of 3 months prior to vedolizumab initiation. Dosing of all therapies was unchanged for at least 1 week prior to the beginning of vedolizumab treatment. There was no dose increase of existing anti-inflammatory therapies until final evaluation at week 14. Steroid tapering till week 14 was done at the discretion of the treating physician. After the start of vedolizumab treatment, no other IBD-related concomitant anti-inflammatory treatment was started until the final evaluation visit at week 14. Vedolizumab (300 mg) was administered to all IBD patients at weeks 0, 2, 6, 10, and 14. All patients in our study were treated by an additional dose of vedolizumab at week 10, as all of them had experienced previous nonresponse to anti-TNF therapy. This strategy was implemented in our institution (University of Erlangen, Germany) to heighten the probability of response to vedolizumab treatment. Blood was drawn in patients at weeks 0 and 14, both times prior to vedolizumab administration. Samples were included in the study after obtaining prior written informed consent from each patient and sample collection was previously approved by the ethical committee and the institutional review board of the University of Erlangen-Nürnberg.

### PBMCs Isolation

From each patient, 20 mL of whole blood were collected in heparin ethylene diamine tetraacetic acid vacutainer tubes before initiation of the study (week 0) and at week 14. The blood was diluted with phosphate-buffered saline (PBS; ratio: 2/3, vol/vol). Thirty milliliters of whole blood/PBS mixture was gently placed over 20 mL of LSM 1077 lymphocyte separation medium (PAA Laboratories, GmbH, Pasching, Austria) using two 50-mL conic tubes sufficient enough for peripheral blood mononuclear cell (PBMC) separation of 40 mL of diluted blood. PBMCs were separated by centrifugation at 805*g* for 20 minutes at 18°C. The isolated PBMCs were washed 2 times in PBS at 652*g*. Pelleted PBMCs were frozen and stored at −80°C until use.

### Physiological Intermolecular Modification Spectroscopy

PIMS is a label-free technology that is able to study protein–protein and protein–solvent interactions in multicomponent solutions. It provides individual real-time dynamic fingerprints of total physiological macromolecular assemblies in tissue or blood in presence and absence of exogenous molecules (drug or drug candidate, peptide or protein).^13^

PIMS is a near infrared (NIR) spectroscopy method based on the modulation of water molecule resonance, which reflects the change in macromolecular conformation due to the interaction of the compound with its target protein, provided this interaction would lead to activation of related pharmacological signaling pathways.^13^ In absence of the latter, there will be no change in water molecule resonance. The NIR bands (at about λ 970–1940 nm) are suited for rapid nondestructive water determination,^20^ all shifting a few nm to longer wavelengths (lower frequency) with strengthening of hydrogen bonding due to shifts from high-density water (increasing collapsed structure) to low-density water (increasing expanded structure).^13,20,21^ PIMS is therefore able to study protein–protein and protein–solvent interactions in multicomponent solutions. It provides individual real-time dynamic fingerprints of total physiological macromolecular assemblies in a tissue in the presence and absence of exogenous molecules (drug or drug candidates, peptides or proteins). It reflects the patient’s molecular capacity to respond to a drug substance and allows to discriminate different subpopulations of patients regarding the capacity to respond to a specific therapy.^13^

**FIGURE 1.**
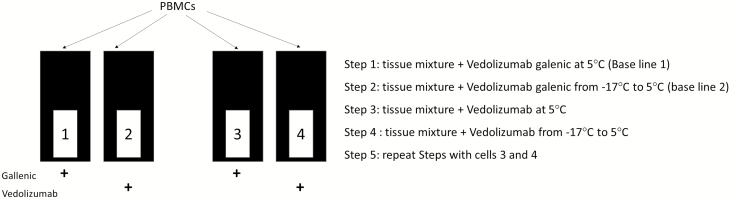
PIMS experimental steps. All experiments are performed using the double beam PIMS instrument. The boxes represent the PIMS cells. All cells contain the mixture of PBMCs. Cells 1 and 3 contain only the excipients (l-histidine chlorhydrate, l-histidine monohydrate, l-arginine chlorhydrate 18, and saccharose polysorbate 80) of vedolizumab, whereas cells 2 and 4 contain vedolizumab in its galenic formulation.

Briefly, PBMC extracts in physiological conditions were frozen at −17°C. Macromolecular spectra are registered as the temperature within the sample raises from −17 to 5°C. The experiments were performed twice. In order to avoid freezing and thawing of PBMCs at the day of the experimental procedure, 4 samples in quartz cells (110 µL volume) were prepared comprising 5 µL patient PBMCs, in 105 µL chilled (4°C) Hanks Balanced Saline Solution (HBSS, Lonza) or 5 µL patient PBMCs in 100 µL of HBSS supplemented with 5 µL of vedolizumab. Samples in absence of vedolizumab were used for determination of the baseline signal in the range from −17 to 5°C. Thereafter, any change from the basal value in the presence of vedolizumab was explored. In the latter, reference control absorption was measured in the absence of vedolizumab (Fig. 1).

As PIMS is based on continues sampling from −17 to 5°C, the entire results from the very same temperature interval were used to calculate the differential molecular oscillation and relative dynamic diffraction.

### NPOT and Proteomics

NPOT is based on a transitory, liquid pH gradient from 5 to 9. The pH gradient is based on a physiological pH allowing protein–protein interactions in mitochondria, acid in lysosome and from the cytoplasm to the nucleus. Proteins complexes from a patient’s tissue will diffuse to their mean zwitterion point (MZP), when they reach a lower metastable energy state. Interaction of the compound or antibody with its target and its related molecular network, changes the initial MZP of the target complex that then consequently diffuses to another MZP. This would induce a molecular crowding and result in formation of nematic heteroassemblies which will precipitate out from the solution. This precipitation is induced specifically in the presence of a ligand, compound, antibody, or any other active agent, in which precipitation is observed.^22–25^

**FIGURE 2.**
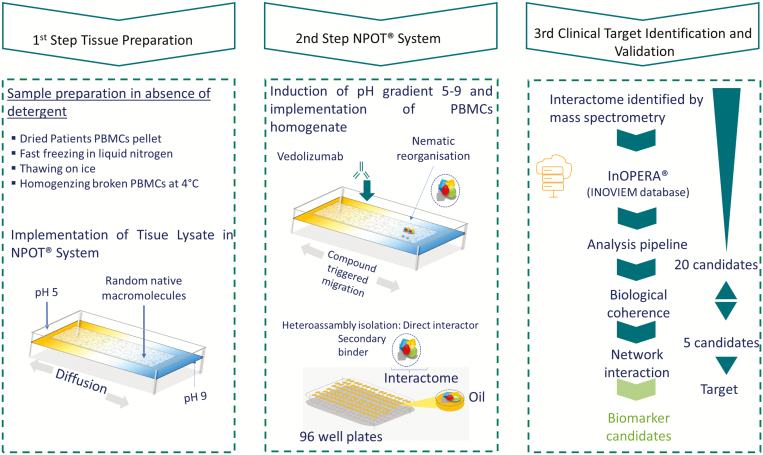
Schematizing NPOT experiment using patients PBMCs and vedolizumab. The first step describes the sample preparation until loading into the NPOT with a pH gradient; the second step illustrates the specific heteroassemblies formation and isolation. The resulting heteroassemblies protein contents are analyzed by LC−MS/MS.

NPOT procedure is also described in detail elsewhere.^14,26–28^ Briefly, PBMC lysates from responder (n = 3) and nonresponder patients (n = 3) at week 14 were prepared under low temperature (4°C) in the absence of any detergent, reducing agent or protease or phosphatase inhibitors. PBMCs were broken through 3 cycles of fast freezing using liquid nitrogen and slow thawing on ice. All probable dilutions and washes were performed in HBSS with equal osmolality, trace elements, vitamins, and salts in concentrations as close as possible to those of the interstitial medium or cytoplasm. Vedolizumab (10^−6^ M) was put in contact with 1 µg of patients PBMC homogenates. The macromolecular assemblies were separated using a differential microdialysis system, based on a transitory pH gradient (5–9) wherein the macromolecules (protein groups) migrate in the liquid phase based on their physicochemical properties. The heteroassemblies were isolated and identified by mass spectroscopy (Fig. 2). The identified proteins were analyzed for their specificity using the following steps: (1) proteins are ranked according to their INOpera score (frequency of protein appearance) in order to identify contaminant proteins. (2) DAVID (Database for Annotation, Visualisation and Integrated Discovery; david.ncifcrf.org) and STRING database (String.org) are used to allow the identification of the enriched activated pathways and the identification of targets.

### Post-NPOT LC–MS/MS

Prior to LC–MS/MS (liquid chromatography coupled to tandem mass spectrometry) experiments, heteroassemblies were solubilized directly in 10 µL of 2D buffer (7 M urea, 2 M thiourea, 4% 3-[{3-cholamidopropyl)dimethylammonio}-1-propanesulfonate, 20 mM dithiothreitol, and 1 mM phenylmethylsulfonyl fluoride). Proteins were precipitated in acetate buffer by centrifugation for 20 minutes at 7500*g*. Thereafter, pellets were digested for 1 hour with trypsin Gold (Promega) at 37°C. Trypsin Gold was suspended at 1 µg/µL in 50 mM acetic acid, and then diluted in 40 mM NH_4_HCO_3_ to 20 µg/mL. The samples were dried in SpeedVac at room temperature. Peptides were purified and concentrated by using ZipTip pipette tips (Millipore Corporation) before proceeding for mass spectrometry analysis through 1 hour LC–MS/MS analyses protocol in an ESI-QUAD-TOF machine. Proteins were identified using Mascot software.

### Statistical Analysis

The experiments, data processing, and analysis were performed in blinded fashion at INOVIEM Scientific. Investigators had no insights into patient’s characteristics or response to treatment. For the purpose of the final analysis, the official clinical database was not being deblinded until medical/scientific review had been completed, protocol violators had been identified (if appropriate), and data had been declared complete. The entire data were transferred to the research institute where they were deblinded and processed for statistical analysis. The deblind procedure was performed simultaneously in presence of the principal clinician investigator, project leader from Takeda Pharmaceutical and principal investigator from Inoviem Scientific. Both clinical results and PIMS results in sealed envelopes were handed to the representative of Takeda Pharmaceutical before the start of the deblinding process. The positive predictive value (PPV) and negative predictive value (NPV) were calculated using the following formulae: PPV = sensitivity × prevalence/sensitivity × prevalence + (1 − specificity) × (1 − prevalence), NPV = specificity × (1 − prevalence)/(1 − sensitivity) × prevalence + specificity × (1 − prevalence).

## RESULTS

### Patients and Clinical Data

Of the 13 vedolizumab-treated Crohn disease patients, 7 were considered as responders and 6 as nonresponders at week 14 of therapy (Table 1). Of the 7 vedolizumab-treated ulcerative colitis patients, 4 were considered as responders and 3 as nonresponders at week 14 of therapy (Table 2).

**TABLE 1.** Clinical Data of the Crohn's Disease Patients (HBI ≥8 at Inclusion) of the Study

			HBI	HBI		
Patient	Sex	Age	Week 0	Week 14	Clinical Result at Week 14	Previous Anti-TNF Therapy
1	F	56	9	7	No response	IFX, ADA
2	M	64	9	1	Response	ADA, IFX
3	F	41	15	10	Response	IFX, ADA
4	F	52	15	10	Response	IFX, ADA
5	F	33	8	2	Response	IFX, ADA
6	F	26	12	4	Response	IFX, ADA
7	M	37	11	1	Response	IFX, ADA
8	M	28	13	3	Response	IFX, ADA
9	F	50	27	25	No response	IFX
10	M	25	8	10	No response	IFX, ADA
11	M	29	8	6	No response	ADA, IFX
12	M	31	20	18	No response	IFX, ADA, CTP
13	F	47	11	10	No response	ADA, IFX
n = 13	7F/6M				7r/6nr	

Thirteen Crohn's disease patients were included in this study. Seven patients were assessed as responders and 6 as nonresponders to vedolizumab at week 14. All patients demonstrated nonresponse (primary or secondary) to previous treatment with an anti-TNF antibody. ADA, adalimumab; CTP, certolizumab pegol; IFX, infliximab.

**TABLE 2.** Clinical Data of the Ulcerative Colitis Patients [Partial Mayo Score (pMS) ≥5 at Inclusion] of the Study

			pMS	pMS		
Patient	Sex	Age	Week 0	Week 14	Clinical Result After Week 14	Previous Anti-TNF Therapy
14	F	63	9	3	Response	GOL, IFX
15	F	34	5	5	No response	ADA, IFX
16	M	26	6	3	Response	IFX, ADA
17	M	52	7	9	No response	IFX
18	F	31	9	0	Response	IFX, ADA
19	F	53	5	5	No response	IFX
20	M	45	7	1	Response	ADA, IFX, GOL
n = 7	4F/3M				4r/3nr	

Seven ulcerative colitis patients were included in this study. Four patients were assessed as responders and 3 as nonresponders to vedolizumab at week 14. All patients demonstrated nonresponse (primary or secondary) to previous treatment with an anti-TNF antibody. ADA, adalimumab; GOL, golimumab; IFX, infliximab.

### PIMS Analysis

**FIGURE 3.**
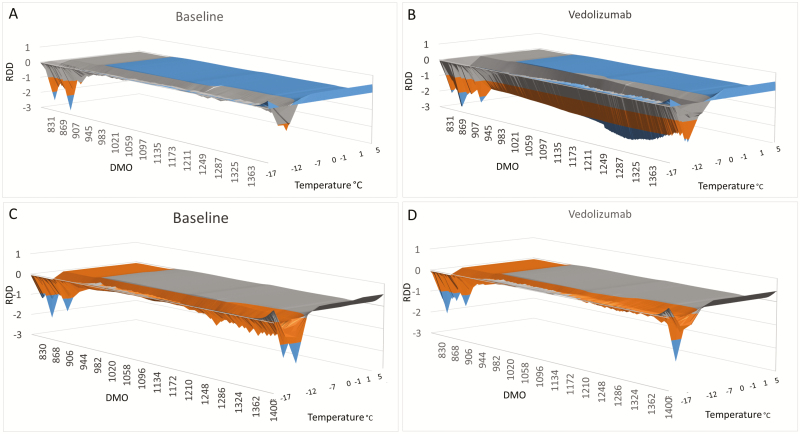
Profile of IMV of an IBD patient nonresponder (A and B) and responder (C and D) to vedolizumab therapy. In nonresponder patients, there was no modulation from the baseline of patients PBMCs macromolecular resonance in presence of vedolizumab. In responder patients, PBMCs macromolecular resonance was modified in the presence of vedolizumab. Note the change in the molecular oscillation in presence of vedolizumab as the temperature rises from −17 to 5°C. For calculating the DMO and RDD, the entire results from −17 to 5°C are taken into account. DMO, differential molecular oscillation; RDD, relative dynamic diffraction.

Profile of the individual macromolecular volume (IMV) during PIMS analysis of an IBD patient nonresponder (A and B) and responder (C and D) to vedolizumab treatment are depicted in Figure 3. These profiles are generated from the continuous sampling of NIR spectra of patient’s PBMC homogenates as the temperature rises from −17 to 5°C. The obtained IMV is the result of the difference between the signal from the test cell and the blank. A negative IMV means that the blank cell has higher molecular resonance compared to the test cell. In nonresponder patients, there was no change in IMV in presence of vedolizumab. This shows the lack of the antibody’s impact on molecular resonance whereas in responder patients, vedolizumab did impact the molecular resonance and therefore altered the IMV.

Prior to deblinding of data, PIMS-treatment predictions were documented for each patient. PIMS analysis of blood taken at week 0 was in accordance with clinical response to vedolizumab treatment at week 14 in 77% of vedolizumab-treated Crohn disease patients (n = 13). In vedolizumab-treated ulcerative colitis patients (n = 7), we found PIMS analysis in accordance with clinical response at week 14 in 100% of patients. The correlation between PIMS prediction and clinical response data was calculated as PPV and NPV for all IBD patients (n = 20) which were 89% and 82%, respectively.

### NPOT Analyses

**FIGURE 4.**
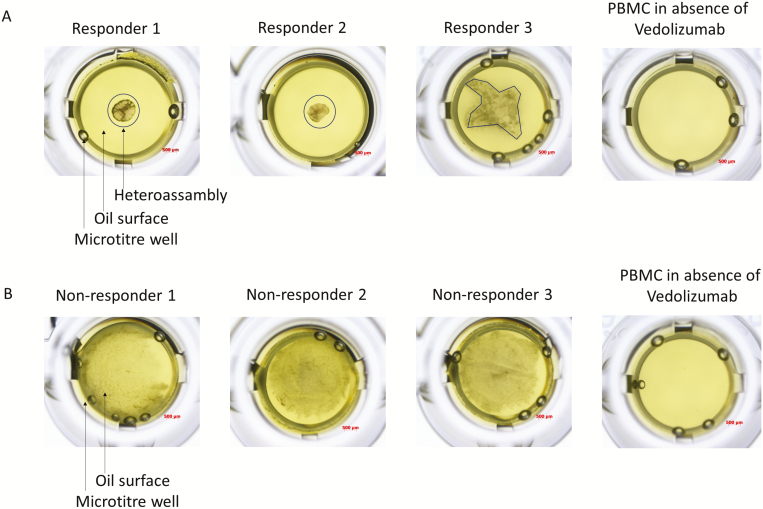
A, Heteroassemblies of 3 vedolizumab responders as assessed by NPOT analysis. Heteroassemblies are depicted by black circles or black lines. Each patient’s PBMC extract was put in contact with vedolizumab and subsequently gave rise to clearly defined heteroassemblies with common reticular morphology and 2 out of 3 patients with symmetric and 1 with asymmetric forms. There are no heteroassemblies formed in the absence of vedolizumab. B, Heteroassemblies of 3 nonresponder patients to vedolizumab. Heteroassemblies are diffused with no distinguishable shape. There are no heteroassemblies formed in the absence of vedolizumab.

For NPOT experiments, 3 IBD patients from the responder and nonresponder group were randomly selected. In the group of 3 responders, there were 1 Crohn disease and 2 ulcerative colitis patients. In the group of nonresponders, there were 2 Crohn disease and 1 ulcerative colitis patients. Blood taken at week 14 of these 6 patients were subjected to NPOT analysis. The obtained heteroassemblies from responders and nonresponders are shown (Fig. 4A, B).

Heteroassemblies from responder patient’s PBMCs homogenates at week 14 were well structured and had distinct forms. Proteomic analysis using LC/MS–MS sequencing identified n = 523, n = 635, and n = 598 proteins, respectively, from each analyzed IBD patient’s PBMC homogenates. The analysis after filtering the frequent hits as well as nonspecific binding, revealed 23 proteins found to be specific for vedolizumab-treated clinical responders. These proteins were subjected to the string database analysis to form the interactome.

**FIGURE 5.**
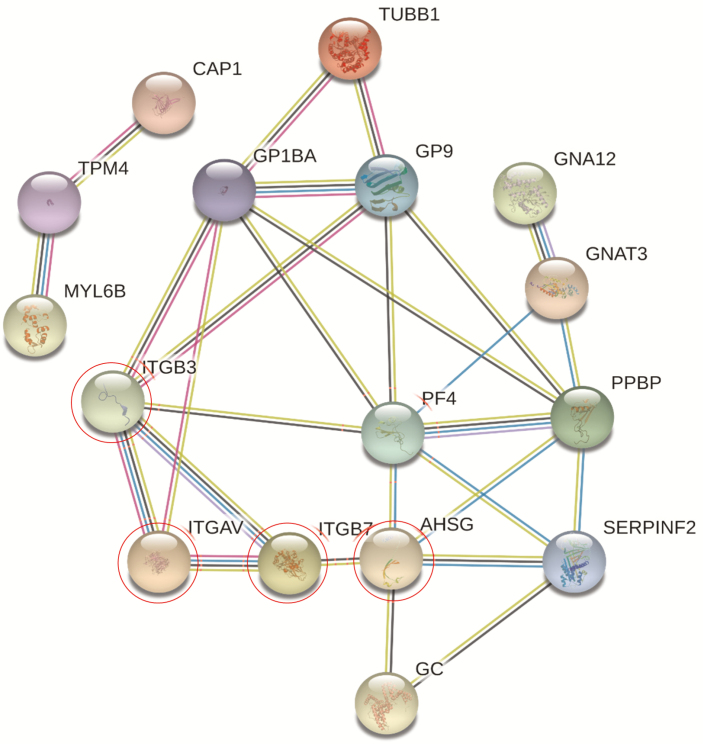
STRING network from responder patients to vedolizumab. NPOT heteroassemblies protein content was analyzed using STRING. The near interactor of signaling pathway is comprised of ITGB7, ITGAV, ITGB3, AHSG, and PF4 (encircled proteins).

Of 23 proteins, 13 build a distinct network. The network consists of the proteins ITGAV (integrin alpha V), ITGB3 (integrin beta 3), AHSG (alpha2-HSglycoprotein), PF4 (platelet factor 4), PPBP (platelet basic protein), GP9 (platelet glycoprotein IX), and GC (vitamin D-binding protein). Of these 7 proteins, ITGAV, ITGB3, and ASHG interact directly with the target ITGB7 (alpha4beta7) whereas the other 4 proteins PF4, PPBP, GP9, and GC interact indirectly with ITGB7 (Fig. 5).

**FIGURE 6.**
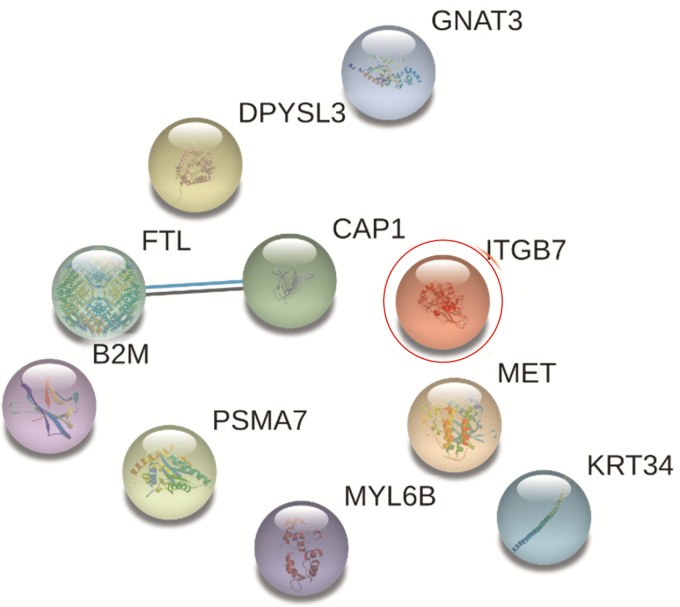
STRING network from nonresponder patients to vedolizumab. There is an absence of interactors and therefore a signaling pathway could not be identified.

Heteroassemblies from nonresponder patient PBMCs homogenate at week 14, contrary to responders, were diffuse and floating on the oil surface (Fig. 6). Proteomic analysis using LC/MS–MS sequencing identified n = 839, n = 589, and n = 569 proteins, respectively, from each IBD patient’s PBMC homogenate. After filtering the frequent hits as well as nonspecific binding, 10 proteins were found to be specific for vedolizumab, which also included its target ITGB7. In comparison to responder patients, no-distinct interaction network could be established (Fig. 6).

## DISCUSSION

The introduction of biological therapies, like the anti-alpha4beta7 integrin antibody vedolizumab, has led to substantial improvements in the treatment of IBD patients. However, only a subgroup of patients responds to the available therapies underlining the dire need for predictive diagnostic procedures to identify therapeutic responders and allows a much needed personalized medicine approach in IBD patients. As anti-TNF antibody exposed IBD patients generally exhibit markedly diminished response rate to any subsequent biological therapy (vedolizumab or ustekinumab), accurate prediction of response is of high therapeutic importance in this difficult to treat patient group.

In our prospective study, PIMS could stratify anti-TNF refractory (primary or secondary nonresponse) IBD patients in regard to vedolizumab clinical response at week 14 with high specificity and good sensitivity (PPV 89% and NPV 82%). NPOT revealed that the functional signaling pathway that was triggered due to the interaction of anti-ITG7 with its target antigen is composed of ITGB7, ITGAV, ITG3, PF4, and ASGH in clinical responders to vedolizumab treatment.

The transient interaction in simple component solutions has been studied using a well-established Rayleigh light scattering method which reflects molecular oscillation.^23,29–34^ Proteins association unfolding, aggregation, and cellular crowding are known to affect both the normal function of the cellular system and are involved in a variety of human diseases.^20,24,25,35–37^

Based on these and the acquired knowledge of light scattering, the novel technology PIMS had been developed. This provides, within the organ of interest, a dynamic fingerprint of the entire macromolecular assemblies for each individual that is reflected through the change in water molecule resonance. The latter can be used for prediction of a treatment efficacy prior to the onset of treatment.^13^ As no protein or macromolecule exists in an isolated form within the cell, the primary target should be in a corresponding quaternary structure in order to trigger a biological activity. Therefore, interaction of an antibody with its target would have a desired pharmacological activity provided it triggers corresponding signaling pathways.

PIMS and NPOT both reveal the functional molecular resonance and the functional singling pathway required for vedolizumab desired activity, as (1) the signaling pathway remains as native as possible, (2) protein–protein and macromolecular interactions are not broken, and (3) molecular plasticity is preserved.

Although in both responder and nonresponder groups, the primary target alpha4beta7 integrin was present in isolated patients’ PBMCs, NPOT could isolate and identify only in the responder group a functional molecular network. A functional molecular network is defined as a network comprised of direct interaction (physical) or indirect interaction (functional) of proteins required to induce and maintain a biological or pharmacological activity. They stem from computational prediction, and from knowledge transfer between organisms.^38–40^ The functional molecular network or any other network was absent in the nonresponder group. NPOT analysis from responder and nonresponder groups revealed the necessity of a dedicated network to achieve clinical efficacy. This could be a plausible explanation for the presence or absence of clinical response in IBD patients treated with vedolizumab. Due to the small sample size of altogether 6 IBD patients, NPOT results must be validated in subsequent studies to rule our possible random effects. Among the identified functional molecular network, 2 proteins were highlighted, vitamin D transporter (CG) and PF4 which are of particular interest as candidates for successful predictive biomarkers. The presence of a CG among the identified proteins is of particularly interest, as vitamin D-binding protein polymorphism has been associated with IBD.^41^ Furthermore, higher vitamin D-binding protein concentrations were significantly associated with disease flares.^42^ Vitamin D normalization has been associated with reduced risks of relapse, IBD-related surgeries, and improved quality of life.^43^ Genetic variants in PF4 have been reported to modulate inflammation.^44^ In a prospective study, the role of serum PF4 as a reliable activity parameter in adult IBD patients has already been successfully assessed.^45^

At the clinical level, these approaches do provide valuable information enabling a more efficient clinical monitoring in a patient-based manner, regarding prediction of response and identification of treatment-specific biomarkers.

The currently performed proof-of-concept study only included a small group of patients and only recorded short-term clinical response to vedolizumab therapy till week 14. Nevertheless, a recently published real-world study in a clinical setting indicated that treatment with vedolizumab beyond week 14 in primary nonresponders did not result in a significant increase in response or remission rates at week 54. Week 14 was therefore proposed as the appropriate time point to assess clinical response to vedolizumab therapy and decide upon continuation or termination of vedolizumab therapy.^46^ We were not able to implement endoscopic evaluations at predefined times in our study. The obtained clinical observations must therefore be accompanied by endoscopy evaluations in further studies, as a stark discrepancy between clinical and endoscopic parameters has been described in both IBD disease entities. In addition, differences in the respective inflammatory burden prior to initiation of vedolizumab therapy could also influence the probability of nonresponse to vedolizumab treatment. Furthermore, severity of disease may also modulate the expression level of the measured proteins. Furthermore, objective biochemical markers of inflammation (fecal calprotectin, or C-reactive protein) should be incorporated in future studies, as they can to some extent reflect the level of mucosal inflammation.

The exact reasons for discontinuation of previous anti-TNF therapy (mechanistic failure or suboptimal dosing, as well as primary or secondary nonresponse) prior to receipt of vedolizumab were not assessed in our study. There were no assessments of anti-TNF trough or antidrug antibody levels to define pharmacokinetic causes for nonresponse to previous anti-TNF therapy.

The obtained results must therefore be validated in further clinical studies with a bigger cohort of IBD patients. These studies must then also include endoscopic assessment at baseline and at the time of assessing response to therapy, and should then also include assessment of biochemical markers of inflammation and long-term follow-up of the patients.

Taken together, this prospective study demonstrates the capacity of label-free based proteomic approaches to predict clinical efficacy of vedolizumab therapy in an individual manner and pave the way for identification of its success predictors. Based on the reported observations and subsequent validation, PIMS and NPOT analysis might be applied in disease management of anti-TNF nonresponders, to decide if subsequent biological treatment with vedolizumab should be initiated or if another therapeutic option (eg, ustekinumab) should be favored instead. In parallel to the preparation of the follow-up study, the clinical values of GC and PF4 as successful biomarkers for vedolizumab therapy will be assessed in the blood of the treated patients. This could open the opportunity to develop a companion test for prediction of vedolizumab efficacy in IBD patients and enable a much needed personalized therapeutic approach, especially in IBD patients failing first line therapy with an anti-TNF antibody.

## Data Availability

The data that support the findings of this study are available from the corresponding author, upon reasonable request.

## Notes

Funding: Conduction of this study has been in part been financed by Takeda Pharmaceutical. This study was also supported by the Deutsche Forschungsgemeinschaft (DFG) to Raja Atreya (RA) (CRC1181 Project C02 and TRR241 Project C02). The Heisenberg Professorship of RA is funded by the DFG.

Conflict of interest: PIMS had been developed and patented by Inoviem Scientific (WO2013139988 (A1)). RA has served as a speaker, or consultant, or received research grants from AbbVie, Biogen, Boehringer Ingelheim GmbH & Co. KG, Dr. Falk Pharma GmbH, Ferring GmbH, GlaxoSmithKline plc, InDex Pharmaceuticals AG, Janssen-Cilag GmbH, MSD Sharp & Dome GmbH, Pfizer Inc., Roche Pharma, Samsung Bioepis, Stelic Institute, Takeda GmbH & Co. KG, and Tillotts Pharma AG. MFN reports research grants and/or personal fees from Abbvie, MSD, Takeda, Boehringer, Roche, Pfizer, Janssen, Pentax and PPD.
